# A Semi-Supervised Approach with Monotonic Constraints for Improved Remaining Useful Life Estimation

**DOI:** 10.3390/s22041590

**Published:** 2022-02-18

**Authors:** Diego Nieves Avendano, Nathan Vandermoortele, Colin Soete, Pieter Moens, Agusmian Partogi Ompusunggu, Dirk Deschrijver, Sofie Van Hoecke

**Affiliations:** 1 IDLab, Ghent University—IMEC, 9052 Ghent, Belgium; nathan.vandemoortele@ugent.be (N.V.); colin.soete@ugent.be (C.S.); pieter.moens@ugent.be (P.M.); dirk.deschrijver@ugent.be (D.D.); sofie.vanhoecke@ugent.be (S.V.H.); 2 Flanders Make—Corelab Decision S, 3001 Leuven, Belgium

**Keywords:** predictive maintenance, health index, remaining useful life estimation, bearing degradation, applied machine learning

## Abstract

Remaining useful life is of great value in the industry and is a key component of Prognostics and Health Management (PHM) in the context of the Predictive Maintenance (PdM) strategy. Accurate estimation of the remaining useful life (RUL) is helpful for optimizing maintenance schedules, obtaining insights into the component degradation, and avoiding unexpected breakdowns. This paper presents a methodology for creating health index models with monotonicity in a semi-supervised approach. The health indexes are then used for enhancing remaining useful life estimation models. The methodology is evaluated on two bearing datasets. Results demonstrate the advantage of using the monotonic health index for obtaining insights into the bearing degradation and for remaining useful life estimation.

## 1. Introduction

Health assessment (HA) and remaining useful life (RUL) estimation of mechanical components is a key task in Prognostics and Health Management (PHM). Accurate PHM models allow improvements in terms of quality, safety, maintenance scheduling, and cost reduction.

PHM techniques are grouped into three categories: model-based or data-driven and hybrid [1,2,3]. Model-based methods use analytical or physical models to approximate the component’s behavior and its degradation. Their main advantage is high accuracy and capability of simulating diverse scenarios, such as operating conditions or different component specifications, without actually having to run a physical experiment. However, real degradation processes are stochastic and can occur within multiple parts of the component, which makes them hard to model. In addition, the increased use of sensors in industries has led to an increased interest in data-driven techniques. Data-driven models use monitoring information to create models that approximate the component behavior and degradation. These models can be either statistical or machine-learning-based. Data-driven models overcome some of the model-based limitations as they require no expert knowledge and can infer degradation and failure from the data without having to specify the failure type. However, their main limitation is the considerable amount of data required; in addition, these models have limitations when exposed to conditions that are not captured in the historical data. Finally, the hybrid techniques consist of a combination of model-based and data-driven approaches with the aim of achieving a better trade-off between accuracy and data requirements.

This paper presents a methodology for constructing PHM models for HA and RUL prediction that handles the two main limitations of data-driven models; more specifically, they are functional in datasets with a small number of samples and features, and able to account for unexpected conditions. In addition, the presented HA model follows a semi-supervised paradigm that requires no labels or expert knowledge. Furthermore, the model training induces monotonic behavior on the outputs.

This paper uses the bearing benchmarks from two test setups, namely the Pronostia setup by the FEMTO-ST institute [4] and the Smart Maintenance Living Lab by Flanders Make and imec [5]. Both test setups emerge from the necessity of having platforms to conduct bearing experiments, generate degradation data under different conditions, and validate prognostic models.

The paper is divided as follows. Section 1.1 presents an overview of related work. Section 2 introduces the setups and datasets. Section 3 outlines the methodology, consisting of the pre-processing, the health index construction, and the model training. Section 4 presents the results and discussion. Finally, Section 6 presents the conclusions.

### 1.1. Related Work

One of the goals of Industry 4.0 is the continuous monitoring of manufacturing components. The trend of the Industrial Internet of Things (IIoT) has increased the number of available sensors in the industry and facilitated the capture of large amounts of data, which in turn has helped the development and deployment of data-driven models.

RUL modeling and prediction usually consists of a feature engineering step followed by a statistical or machine learning model. Feature engineering steps include, among others, generating time-based and frequency-based features, feature selection, and dimensionality reduction. Feature selection is often done by evaluating monotonicity and trendability, which are intuitive choices as they can be strongly correlated to degradation over time in systems that do not experience recovery [3,4,6]. Alternatively, some methods focus on modeling an auxiliary target that serves as a health index (HI) [3]. Usually, the HI is a dimensionless value that represents the condition of the component. The HI can be constructed based on expert knowledge or statistical models and designed to satisfy specific requirements such as being bounded or monotonic. In some cases, the HI serves as an intermediate step before RUL estimation [3].

Concerning a HI construction, previous work has focused on obtaining smooth indicators by processing the input signals and selecting a feature or combination of features that match the expected degradation behavior. Zhang et al. use single value decomposition (SVD) to reduce multivariate sensor data, and then preserve only a limited number of components [7]. The components are projections of the data that capture the main changes over time and are used as HI. Mosallam et al. present a method that uses multiple time-based features and Empirical Mode Decomposition (EMD) residuals as HI [8]. The proposed HI is noise-free and able to detect early degradation. However, the results only include selected bearings from the Pronostia dataset. Moreover, transformations in the data can be used to induce monotonicity in the features. The cumulative sum of features is used in combination with a particle filter for RUL estimation [9]. Although HIs are interpretable and inherently correlated with time, they are unbounded, which usually leads to sharp changes towards the end of life of the component. Previous work has also proposed models with bounded HI curves. Wu et al. evaluated four possible degradation curves for the Pronostia dataset [10]. The intermediate HI is modeled with different candidate functions, such as linear, inverse hyperbolic tangent, quadratic, and parabolic. Their results point to the inverse hyperbolic tangent being the best HI model. Although the results are good, it is expected that a free form function could bring better results.

Although HIs are indicative by nature, their evaluation is hard to assess as there is no reference value to compare with. Therefore, it is important to assess how informative the HI is in PdM tasks, such as by evaluating on failure prediction or end of life estimation tasks. In this work, the proposed HI is evaluated on its own as a tool for visual information and condition monitoring and also used to derive additional features for RUL estimation.

The RUL estimation is closely related to the HI. However, in this case, the target is the actual time before the component breaks. The winners of the IEEE 2012 PHM Data Challenge proposed three models: a moving average of the spectral kurtosis in combination with Bayesian Monte Carlo estimation, which offers RUL estimations as histograms; a second model that uses a set of extracted features that then are reduced using PCA and modeled using least squares support vector regressor (LS-SVR); and, finally, a method where anomaly detection (AD) is used in combination with curve fitting to approximate degradation curves [11]. The AD approach is of particular interest as they use anomaly counting to identify degradation stages. In some cases, the HI is used as an additional feature for the RUL model. For example, an intermediate HI target is used to identify similar behaviors and then perform RUL estimation [12]. However, no specifications of the RUL model are available, and it only presents results for a single bearing of the Pronostia dataset. Finally, Fractional Lévy Stable Motions have been used in combination with clustering methods to create multi-mode models in which the different degradation stages can be incorporated [13]. This is of particular interest as previous work has pointed out how component degradation can be subject to changes in trends over time, and it can be especially affected by maintenance or stop events.

More recently, deep learning techniques that make use of spatial information, such as Convolutional Neural Networks (CNN), or time dependencies, such as Recursive Neural Networks (RNN) and Long Short-Term Memory (LSTM), have been used. Pretrained CNN are adapted using a genetic algorithms approach for the RUL estimation of punch tools [14]. The LSTM architecture is used to predict a HI and RUL [15]. The HI target corresponds to the percentage of time before failure. However, a HI based on time may be prone to information leakage and overfit, as it is inherently correlated to the target value. LSTMs can also predict directly the RUL without the need of HIs [16]. The approach relies on detecting the degradation onset and only then estimating the RUL. Using LSTMs gives the advantage of capturing temporal dynamics. In addition, restricting the prediction of RUL to the time after onset reduces the bias in long runs. However, only the results for a single bearing are presented. Finally, an LSTM model with interpretable parameters is proposed by Kraus et al. [17]. This method is evaluated on a different dataset than the one presented here, but points towards the importance of interpretable models. Finally, more advanced techniques such as graphical convolutional networks have been used as feature extractors and combined with a temporal CNN for RUL prediction [18]. This last approach is highly relevant as it shows how intermediate feature mapping proves beneficial for RUL models. The authors select a subset of temporal features and obtain comparable results to the state of the art in the PHM challenge. However, this work only considers temporal features.

Furthermore, imposing monotonicity has been investigated for problems where the target variable needs to be monotonic with respect to one or multiple inputs [19]. This is achieved in multiple ways, such as constraining a more flexible function to be monotonic, post-processing the output to remove violations, modifying the penalty function of the algorithms, or by pre-processing the variables and targets to impose monotonicity before training. Most of the works concerning RUL estimation in bearings focus on creating and selecting features based on a monotonicity criterion [6,8,9,15,16,20,21,22] or post-processing the predictions to fulfill monotonicity [23]. Similar to this approach, structural learning has been used to leverage temporal relations in time series data to induce consistency between continuous predictions [24] and impose monotonicity [25].

To summarize, most of the published works use feature engineering to generate a set of variables that are close to monotonic and have a trend correlated with time. However, the obtained features may not necessarily be monotonic, can contain great fluctuations, and tend to have abrupt changes towards the end of life. In addition, the use of bounded targets has not been investigated in detail, which can help for generalization purposes and offers the possibility for adequate thresholding and calibration. The proposed methodology overcomes most of the previously found limitations.

The presented HI-RUL method consists of three stages. First, the HI targets are generated offline using structural learning. These targets are, by design, bounded and monotonic. Second, a HI model is trained across all bearings for better generalization. The HI predictions are used to detect degradation onset and reduce the datasets to the degradation phase for the RUL modeling. In addition, a set of features derived from the HI are created to keep track of anomalous events. Finally, the RUL model is trained on the reduced datasets using a set of statistical and model-based features. The RUL models are further improved by adding the HI-derived features.

More importantly, the methodology specifically (1) removes the need of assuming the degradation curve mode and instead infers one based on the time behavior of the features, such as trendability and smoothness; (2) provides HI values that are easy to interpret and able to detect events of interest, such as sudden changes and the degradation onset; (3) generalizes the offline HI curves using a machine learning model to allow predictions on unseen data, and (4) leads to HI values that are proven to improve the RUL predictions.

## 2. Setups and Datasets

### 2.1. Pronostia Dataset

The IEEE PHM 2012 Prognostic Challenge dataset comes from the Pronostia platform [4]. The dataset contains recordings of vibration data and other operational parameters, such as load and shaft rotational speed. The bearings are tested under different loads and speeds. Table 1 summarizes the conditions and the number of datasets available. Each dataset starts with a bearing, which is run until failure. The total running time ranges from 1 up to 7 h. The dataset contains no information concerning the failure mode of each bearing. It is only reported that each degraded bearing contains defects in all the components: balls, rings, and cage. Vibrations are measured with two accelerometers placed at 90${}^{\circ }$ orientation to measure both vertical and horizontal vibrations. The sampling frequency is 25.6 kHz. Temperature is also recorded for some bearings. Although the temperature is a good metric for condition monitoring, it is not included in this analysis as not all datasets include it. There is no information available concerning the failure or stop condition but previous work has pointed out that, from visual inspection, it seems to be reaching a vertical acceleration of either 20 g [26] or 30 g [21].

Predicting RUL for the Pronostia dataset is considered challenging due to the small learning set and the high variation in running times. In addition, the creators of the challenge report that theoretical models based on frequency signatures, such as inner and outer race frequencies, do not provide good results. Frequency-based models focus on analyzing only a limited amount of frequency bands, but, in practice, degradation occurs on multiple components and therefore has a complex signal across multiple frequencies [4].

### 2.2. Smart Maintenance Living Lab Dataset

The Smart Maintenance Living Lab (SMLL) is an open test research platform that aims to support the adoption of condition monitoring technologies [5]. The platform consists of a fleet of seven identical drivetrain setups that perform accelerated lifetime tests on bearings. The fleet offers two advantages: first, it allows faster data collection; second, identical drivetrain systems can have variability. Therefore, it offers the opportunity for training and evaluating robust models [5,27].

**Figure 1:**
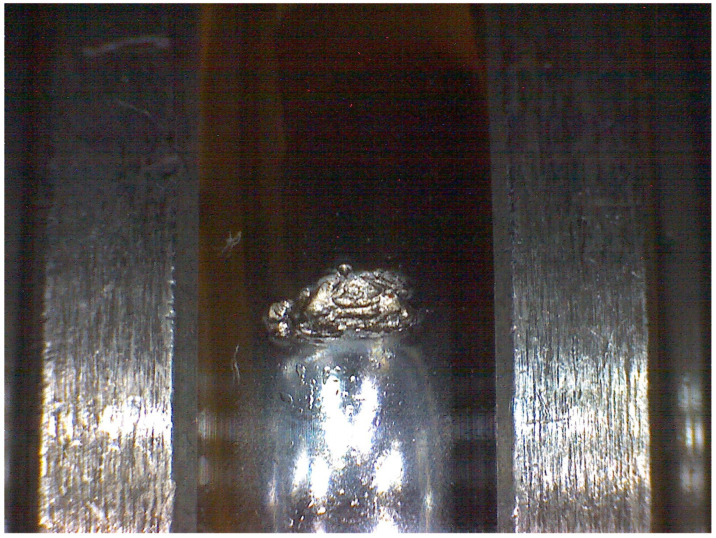
Bearing A43.

**Figure 2:**
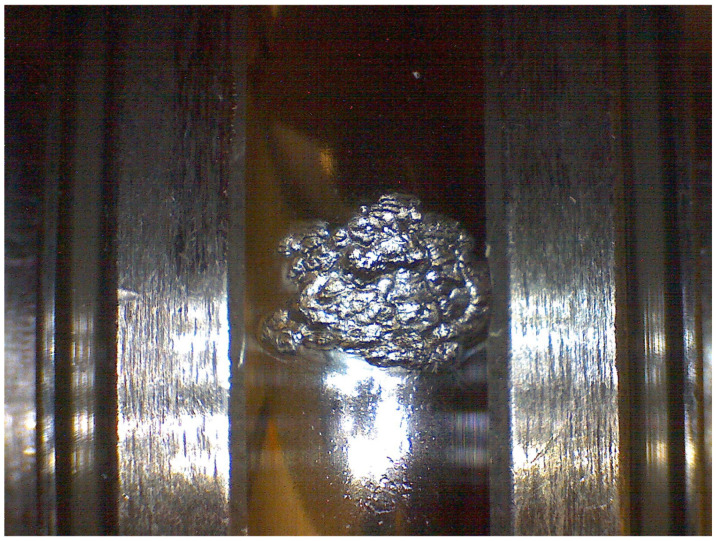
Bearing A47.

The dataset consists of tests under seven different setups with the same testing conditions. There are in total 43 tested bearings, of which 38 are indented and 5 are healthy. Indented bearings are meant to accelerate degradation. The indentation diameters are within 400 ± 25 μm. The indent is small enough to allow the bearing to be considered healthy at the beginning of the test but there is sufficient damage to guarantee that the degradation onset occurs within some hours. The accelerometer data are sampled at 50 kHz. The dataset contains multiple stop conditions, and multiple speed and load conditions; in order to simplify the analysis, only a subset of the data is considered. The subset is composed of bearings that were tested under the same speed and load, namely 2000 rpm and 9 kN, and the same stopping conditions. In the case of healthy bearings, this is defined as the moment when the temperature stabilizes and at least a period of two hours has passed. For the indented bearings, the stop condition is 30 min after the peak to peak vibrations exceed a magnitude of 5 g. Figure 1 and Figure 2 show examples of the final bearing condition after the accelerated life tests. Table 2 summarizes the dataset.

The vibrations are measured using accelerometers with a sensitivity of 100 mV/g with ±5% response deviation in the frequency range of 0.5 Hz to 5 kHz. The sampling rate is 50 KHz and each sample point corresponds to one second of captured data. Further details of the setup can be found in the work of Ooijevaar et al. [5].

The dataset features are derived from the raw vibration signal and based on physical properties or statistics. The raw signal is first filtered and demodulated as described in Ooijevaar et al. [5,28]. Then, the features of interest are calculated on windows of one second. Statistical features include the root mean square (RMS) and kurtosis. Physical features include the ball pass frequency of the inner race (BPFI), the outer ring (BPFO), and the ball defect frequency (BDF). These features correspond to the magnitude at specific frequencies from the spectrogram representation obtained by using the Fast Fourier Transform (FFT) method. Table 3 summarizes the features and presents the corresponding equations. These features are selected based on previous results in which a larger set of 83 features were generated and filtered using forward feature selection.

## 3. Methodology

**Figure 3:**
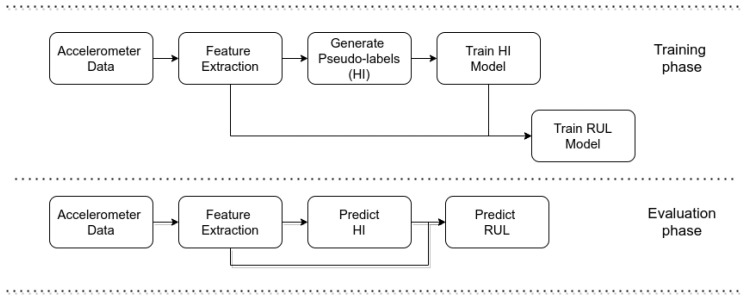
Overview of the pipeline.

This section presents the methodology to generate and validate the HI and the RUL estimation models. Figure 3 presents the pipeline of the proposed methodology, which is divided into a training and testing phase. For training purposes, the pipeline consists of (a) the feature extraction, which is described in Section 3.1, followed by (b) generating the offline HI targets using semi-supervised learning, which is described in Section 3.2, (c) the HI modeling, which is described in Section 3.3, and (d) the RUL modeling, which is described in Section 3.4. The pipeline evaluation is the same, with the only difference being that the offline HI targets are not generated. Finally, this section ends with the specifications for the parameter tuning and validation in Section 3.5.

### 3.1. Feature Extraction

In order to obtain comparable results between both datasets, the features present in the SMLL dataset are calculated for the raw signals of the PHM dataset. Common statistical features, including RMS, kurtosis, peak-to-peak value, etc., are calculated from the raw vibration signals. The physics-based features are calculated from the enhanced envelope spectra after removing disturbances from other machine components such as shafts and gears [29]. Since the PHM Pronostia dataset consists of signals of short duration, the strength of the model-based features cannot be fully exploited. Despite this, the statistical features (i.e., kurtosis, RMS) are still valid, and the model-based features are informative.

### 3.2. Semi-Supervised Health Index

**Figure 4:**
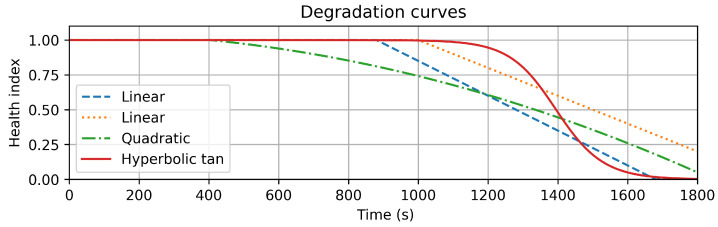
Possible degradation curves with different degradation onset times, degradation rates, changes in the degradation rate over time, and lifetime ends.

To obtain better RUL predictions, an intermediate health index is introduced. This index is related to the bearing condition and inferred from the data. The index should be monotonic with respect to time, without using the time itself as a feature to avoid overfitting. In addition, it should be shape-free so as to not impose assumptions on the degradation rate. The modeling is based on the assumption that, during operation, a bearing condition cannot recover: once damage has initiated, the bearing will continue to degrade until failure. In addition, the index is bounded to allow interpretability and calibration. There exist many functions that can fulfill these requirements. Figure 4 shows some examples. The important elements of the desired function are as follows: an initial flat stage where no degradation has been detected, followed by the degradation onset, and ending with a trend that describes the degradation according to flexible shapes.

The proposed health index is based on structural learning [24,25] and pseudo-labels [30]. Under the pseudo-label paradigm of Lee et al., only a small amount of samples have labels and a large amount of samples have unknown labels. The samples with unknown labels are assigned an initial pseudo-label based on their similarity with labeled samples. The training then iterates between (1) updating the classifier using the pseudo-labels and the known labels, and (2) updating the pseudo-labels using a given classifier. This approach has commonly been used with large image datasets where a number of images are manually labeled and the rest are unlabeled [30,31]. The structural learning approach is a variation of the pseudo-labels by Lee, in which the pseudo-labels are applied on a whole group instead of individual instances, which facilitates the application of constraints. For example, if a set of samples is ordered in time, one can impose a loss function that penalizes the models for having consecutive samples with different labels [24]. In the case of time series with continuous spaces, the constraint can instead be monotonicity [25]. Finally, although the original pseudo-label publication and related ones focus on deep neural networks [24,30], the procedure is applicable for any machine learning algorithm.

Algorithm 1 presents the pseudo-code for generating the pseudo-labels. It consists of the following:

- Lines 1–2. A small fraction of samples at the beginning and end of the dataset are labeled with ones and zeros, respectively, where one represents a healthy status and zero a completely degraded bearing.
- Line 3. The model is trained using only the labeled samples.
- Line 4. The first pseudo-labels are predicted for all the samples.
- Line 5. The time of degradation onset *t* is found by solving Equation (1).
- Line 6. The new targets $\hat{y}$ are assigned following Equation (2).
- Lines 7–11. The iteration loop begins, which is similar to the previous lines (3–6), with the difference that the model is trained using the pseudo-labels $\hat{y}$ instead of only the original labeled samples.
- Line 12. The output of the algorithm is the last pseudo-labels after convergence or reaching the number of iterations.

 **Algorithm 1:** Pseudo-label assignment

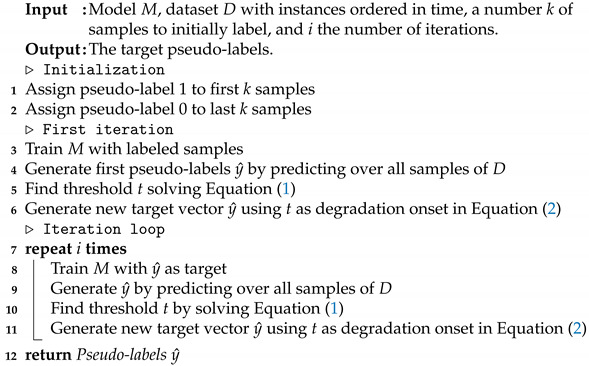



Finding the threshold *t* is similar to solving a logistic regression. Equation (1) contains the optimization problem, where $\hat{y}$ is the pseudo-label vector with *N* instances.

(1) $$t=\mathrm{argmax}\sum_{n=1}^{t}\log\left({\hat{y}}_{n}\right)+\sum_{n=t+1}^{N}\log\left(1-{\hat{y}}_{n}\right)$$

(2) $$\begin{array}{c} \begin{array}{c} f\left(i\right)=\left\{\begin{array}{cc} 1 & \;\mathrm{if}\;i\leq t\\ \phi (x_{i},\theta ) & \;\mathrm{if}\;i>t \end{array}\right.\\ \;\\ \hat{y}=\varphi \left(f\right) \end{array} \end{array}$$

**Figure 5:**
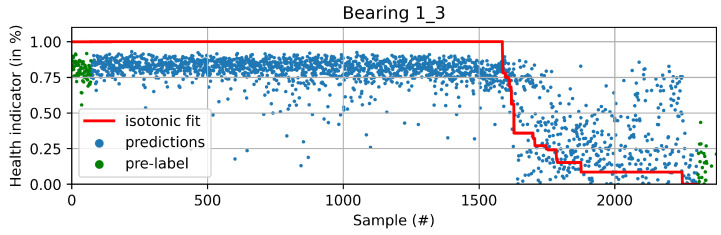
First iteration of the offline HI algorithm. The predictions correspond to the outputs of the model after training for one iteration with only labels for the first and last samples (labeled in green).

**Figure 6:**
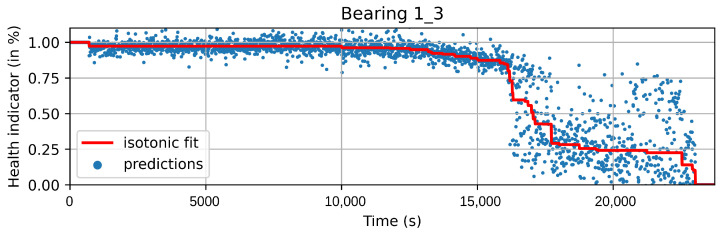
Model predictions and best isotonic fit after 20 iterations of the HI algorithm. Highlighted in red is the last fit of the isotonic, which is used for the generalized model.

The new pseudo-labels are generated according to Equation (2). Instances before *t* are considered healthy and given a value of one. Instances after *t* are considered to be in degradation. In this case, *t* is the threshold index, ϕ is the prediction of the machine learning model with parameters θ, and $x_{i}$ is the instance *i* of the training dataset. The pseudo-labels $\hat{y}$ are generated according to the best fit of the isotonic regression φ over *f*. Equation (3) shows the isotonic regression as an optimization problem, which corresponds to finding the monotonic curve $\hat{y}$, which summarizes *f* with the smallest quadratic error. The solution can be found using the adjacent violators algorithm [32]. Figure 5 and Figure 6 show examples of the iteration process; in Figure 5, the first fit iteration shows the data used to train the model (green), the predictions obtained after the first fit (blue), and the best fit of the isotonic regression algorithm (red). Figure 6 shows the fit after 20 iterations.

(3) $$\begin{array}{c} \begin{array}{c} \hat{y}=\mathrm{argmin}\sum_{i=1}^{N}{\left(f_{i}-{\hat{y}}_{i}\right)}^{2}\\ \mathrm{where}\;{\hat{y}}_{1}\leq {\hat{y}}_{2}\cdot \cdot \cdot \leq {\hat{y}}_{N} \end{array} \end{array}$$

**Figure 7:**
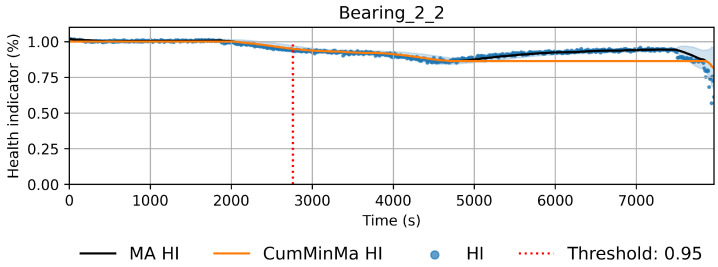
HI of bearing 2_2 of Pronostia dataset. The HI seems to recover gradually after time step 5000. In order to account for these types of events, the cumulative minimum is extracted as a feature.

**Figure 8:**
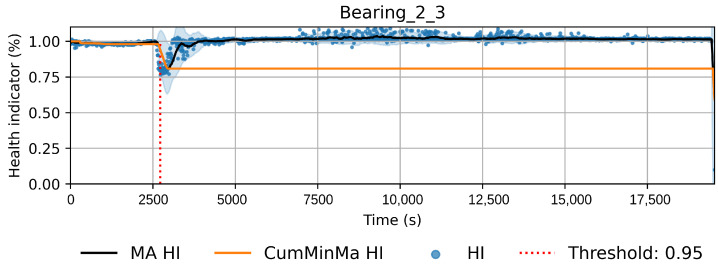
HI of bearing 2_3 recovers from a drastic event around time step 2500. In order to account for these types of events, the cumulative minimum is extracted as a feature.

**Figure 9:**
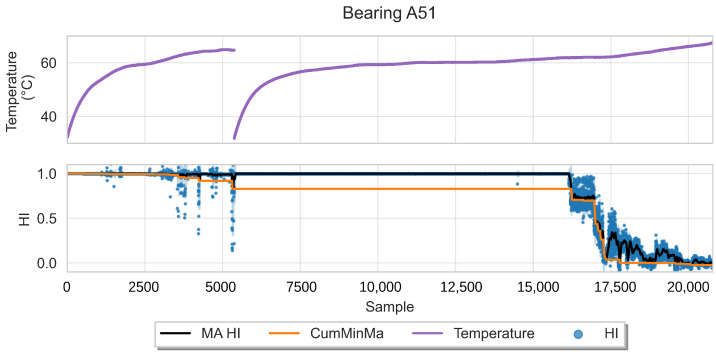
Temperature and HI of bearing A51 of the SMLL dataset. A drop in the HI is detected before the machine is stopped. After the machine is restarted, the HI seems to have recovered.

**Figure 10:**
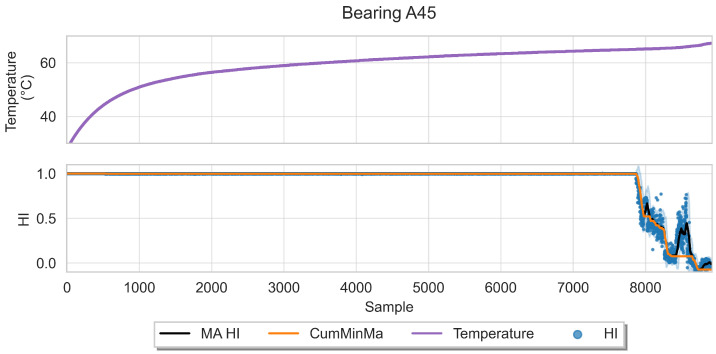
Temperature and HI of bearing A45 of the SMLL dataset. The HI seems to recover over time without it being related to a stop in the machine.

This approach induces semi-monotonic offline indicators. However, there are two scenarios in which the bearing condition may seem to recover in the base features and which can cause discrepancies in the HI. The first scenario, namely the pseudo-recovery, occurs when the initial damage causes a sharp deviation in the features, which then fades away as the bearing initial damage smooths [3,33]. The second scenario occurs when machines stop for some reason, i.e., finishing a production batch or maintenance of other components. The stop causes changes in the mechanics of the bearing, i.e., temperature decreases, which in turn can create the effect of a component recovering. This scenario only concerns the SMLL dataset, where some of the indented bearings were run over a period of some hours before stopping and continuing over the next day(s). During the offline generation of the HI, these scenarios do not pose a problem; however, during the online evaluation, they may cause models to give optimistic predictions. Therefore, two aggregated features are derived from the HI: the moving average (MA), which reduces the variance, and the cumulative minimum of the moving average (CumMinMa), which keeps track of such changes. Figure 7 and Figure 8 show examples of pseudo-recovery, in which the online HI recovers over time after initial damage in bearings from the PHM dataset. Figure 9 shows an example of pseudo-recovery after a stop and Figure 10 shows an example of pseudo-recovery after an anomalous event in bearings from the SMLL. In all these cases, the MA reduces the high variance during inference, and the CumMinMa keeps track of pseudo-recovery events.

### 3.3. Health Index Model

In the previous section, the HI generation was described as an offline process in which the information of the entire run is available. In order to generalize this step and make it applicable to unseen information, a HI model is trained. The model uses the extracted metrics as features (see Table 3) and the generated offline HI as targets.

The selected machine learning model is the Stochastic Gradient Descent (SGD) regressor, which uses the gradient descent algorithm on random subsets of the samples to approximate the gradient of the whole dataset [34]. This algorithm is chosen due to its interpretability as well as the smoothness in its predictions; however, notice that any other machine learning or statistical method is applicable.

### 3.4. Remaining Useful Life Estimation Model

The RUL estimation model follows a similar approach to the one of the HI model, with the difference that the target is the logarithm of the remaining useful lifetime. The model uses the extracted metrics (see Table 3) as features, in addition to the HI and the two HI-based features, namely the MA and the CumMinMa.

**Figure 11:**
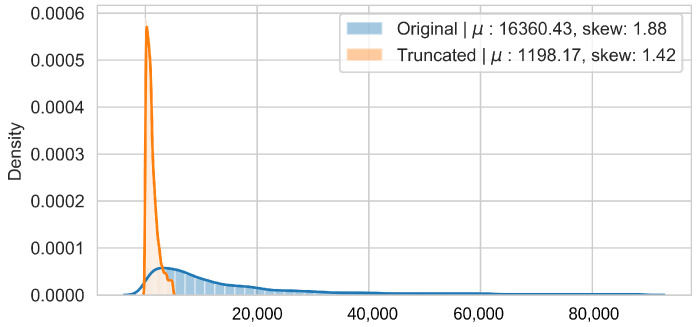
Distribution of RUL values before and after truncation for the SMLL bearings.

**Figure 12:**
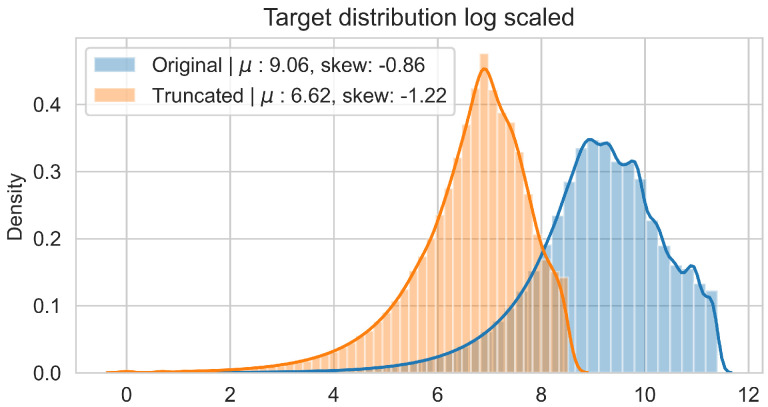
Distribution of log-transformed RUL values before and after truncation for the SMLL bearings.

**Figure 13:**
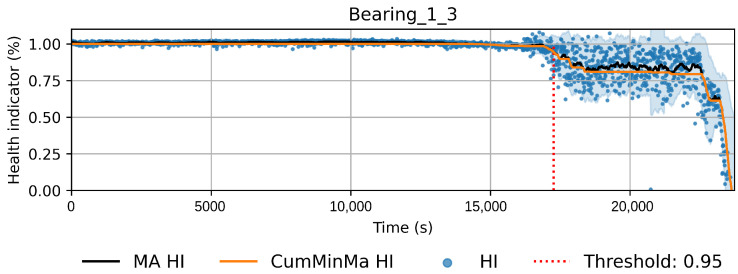
Health index predictions for bearing 1_3. Intervals as the +/−1 running standard deviation.

**Figure 14:**
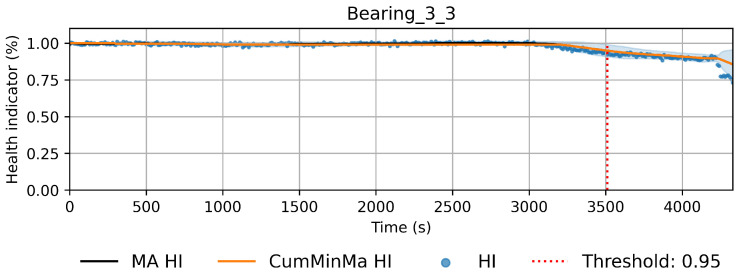
Health index predictions for bearing 3_3. Intervals as the +/−1 running standard deviation.

**Figure 15:**
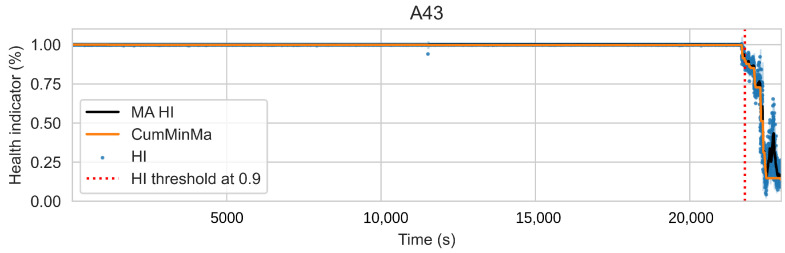
Health index predictions for bearing A43. Intervals as the +/−1 running standard deviation.

**Figure 16:**
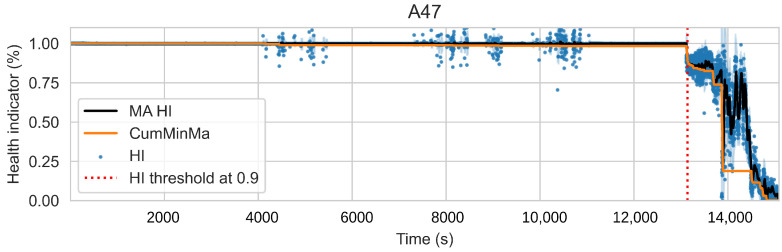
Health index predictions for bearing A47. Intervals as the +/−1 running standard deviation.

In order to reduce the impact of long runs, two measures are taken. First, the RUL model is trained only on data of the degradation phase, which is determined as the moment after which a pre-defined HI threshold is surpassed. The HI threshold is 0.95 for the PHM dataset and 0.90 for the SMLL dataset. These thresholds were empirically defined based on visual inspection of the offline HI over the training datasets. Second, the target is the logarithm of the RUL, which helps to reduce the impact that long tails have in modeling the target value. Figure 11 compares the distribution of the full dataset and the truncated version. The truncation of the data has a considerable impact on reducing the skewness. Figure 12 compares the distribution of the log-transformed RUL before and after truncation. The skewness is further reduced but, in this case, flipped to a negative long tail, which induces the distribution mean towards smaller values. The degradation onset detection is shown in Figure 13 and Figure 14 for the Pronostia dataset and in Figure 15 and Figure 16. During prognosis, RUL predictions are not made unless the HI has passed the defined threshold.

### 3.5. Training and Validation

This section explains the procedure to split the data to perform training and validation. The pipeline consists of three models, namely the offline HI generator, the online HI model, and the RUL model.

#### 3.5.1. HI (Offline)

The offline HI is a weak learner used for generating the health indices offline and does not involve any parameter tuning. The goal is to produce a curve that loosely approximates the progression of the input features without leading to overfitting. A fixed set of hyperparameters is selected and the last iteration of the generated HI is visually examined. Table 4 shows the parameters.

#### 3.5.2. HI (Online) and RUL

For the prognostic HI model and RUL model, the parameters are tuned using Bayesian optimization on the cross-validation error and a limit of 20 iterations. Table 4 shows the parameter search space.

Parameter tuning and evaluation are done in different ways for each dataset.

1. The Smart Maintenance Living Lab dataset is approached with leave-one-group-out cross-validation (LOGO-CV). To avoid problems with serial correlation, each bearing is assigned a unique group. This approach guarantees that the complete data of a bearing are either in the train or validation but not in both stages for a fold.
2. The Pronostia dataset is approached as in the original competition, where only the learning set is used for training and parameter tuning, and evaluated on the remaining bearings (see Table 2). LOGO-CV within the 6 training bearings is used for parameter tuning.

The RUL results are compared against two baselines. The first baseline model, referred to as the full baseline, is trained with only the extracted features from Table 3 and uses the complete training data. The second baseline, referred to as the truncated baseline, uses the same features but is trained only on the left truncated datasets according to the HI threshold. To create a comparable error, the evaluation in all cases is done over the remaining samples of the truncated test set. Notice that, in practice, the truncated baseline requires the HI model but does not use the HI-derived features. The full baseline is used to evaluate the benefits of reducing the datasets to the degradation. The truncated baseline is used to evaluate the benefits of the HI-derived features.

## 4. Results

This section presents the results of the PdM indicators, namely the HI and RUL. Section 4.1 discusses the general observations of the HI as a PdM indicator and Section 4.2 considers the RUL results for each of the two datasets.

### 4.1. PdM Indicators

The obtained HIs can capture the healthy status, degradation onset, and damage progression of most bearings. Figure 13 and Figure 14 show examples of the online HI obtained for Pronostia bearings, and Figure 15 and Figure 16 for SMLL bearings. Although the HIs are informative in most cases, there are instances in which the offline HI may not be reliable. For example, when failure onset occurs close to the end of the test, there are few left samples that include a change in the data. This in turn causes the offline HI generator to produce incorrect points for degradation onset and will ultimately propose flat curves with only ones or zeros. This is the case for several of the bearings of condition 2 in the PHM dataset. The sudden failure has previously been reported in the literature and in many cases confirmed as hard to detect and model [4,10,35]. This may be partially alleviated with the use of richer features.

**Figure 17:**
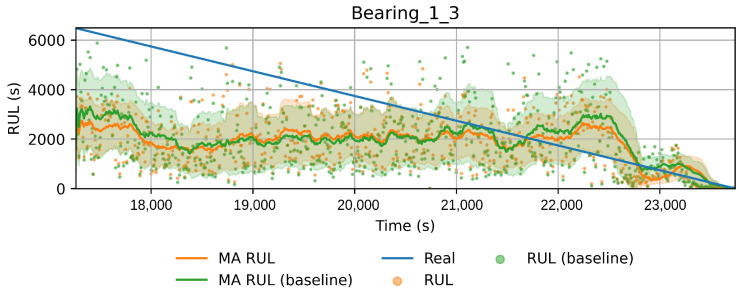
RUL estimation for bearing 1_3. Comparing the benchmark (green) and the proposed method. Intervals as the +/−1 running standard deviation.

**Figure 18:**
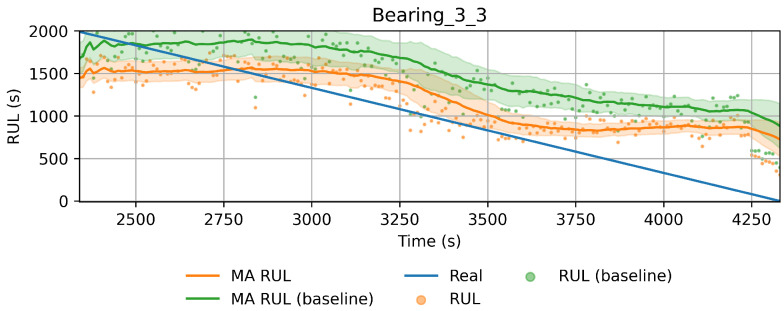
RUL estimation for bearing 3_3. Comparing the benchmark (green) and the proposed method. Intervals as the +/−1 running standard deviation.

**Figure 19:**
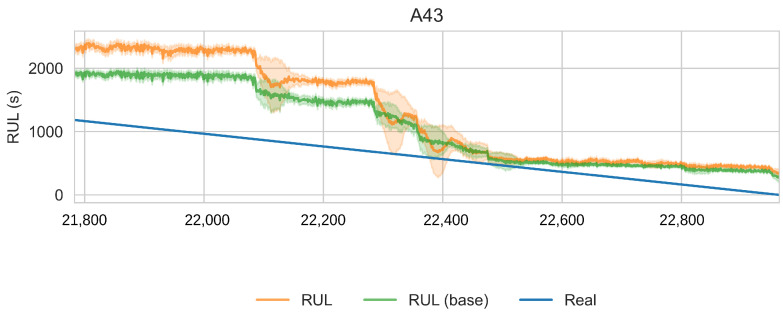
RUL estimation for bearing A43. Comparing the benchmark (green) and the proposed method. Intervals as the +/−1 running standard deviation.

**Figure 20:**
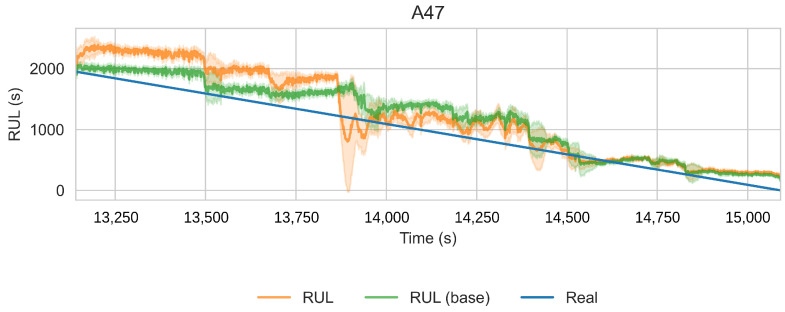
RUL estimation for bearing A47. Comparing the benchmark (green) and the proposed method. Intervals as the +/−1 running standard deviation.

Concerning the desirable properties of HIs, the following was found:

1. Monotonicity. Although the offline target for the HI is strictly monotonic, during evaluation, the predictions contain high variance and pseudo-recovery. This is likely caused by inherent variance from the input variables and the damage smoothing effect. To reduce their impact, the moving average and the cumulative minimum of the moving average of HI are passed as additional features to the RUL model. Figure 13, Figure 14, Figure 15, Figure 16, Figure 17, Figure 18, Figure 19 and Figure 20 show examples of how the moving average reduces the variance and the cumulative minimum keeps track of the initial shocks.
2. Stable regions. The HI shows stable regions within the degradation process. This is similar to what other works have found, where stable regions are used to establish failure stages and estimate expected RUL [13,35]. However, this comes with a trade-off as long stable regions can produce high variance in the RUL estimates. This scenario is partially compensated by the presence of the original features, which are not strictly monotonic.
3. Performance on healthy bearings. The healthy bearings from the SMLL dataset were evaluated with the final HI model and none of them reported a significant drop in the moving average of the HI (window length of 30 samples). The smallest HI values recorded across the healthy bearings were >0.94.
4. Interpretability. The online HI predictions correctly report the healthy stage, detect the degradation onset, and describe degradation over time without the risk of time correlation bias. These features can be used as a tool for condition monitoring.
5. Enhanced features. The HI-derived features are relevant for the RUL estimation, which will be discussed in more detail in the following section. Their effect seems to be more significant in the PHM models.

### 4.2. Remaining Useful Life

The RUL evaluation is done in the original time scale for interpretability. Two metrics are evaluated: the mean absolute percentage error (MAPE) defined in Equation (4) and the root mean squared error (RMSE) defined in Equation (5). The MAPE calculation includes a constant term *c* to avoid divisions by zero. MAPE is a metric that gives more importance towards the smaller target values, which fits the task as more accuracy is desired towards the end of the components’ lifetime. Moreover, MAPE penalizes overestimations more strongly than underestimations.

(4) $$\mathrm{MAPE}=\frac{1}{n}\sum_{t=1}^{n}\left|\frac{\left(y_{t}+c\right)-\left({\hat{y}}_{t}+c\right)}{\left(y_{t}+c\right)}\right|$$

(5) $$\mathrm{RMSE}=\sqrt{\sum_{i=1}^{n}\frac{{\left({\hat{y}}_{i}-y_{i}\right)}^{2}}{n}}$$

The following two sections explain in detail the obtained results.

#### 4.2.1. PHM Data Challenge

Table 5 presents the coefficients for PHM data challenger models. Table 6 reports the performance of the RUL model over the run after the HI threshold is surpassed or at least the last 200 samples in cases where the HI did not reach the threshold (0.90). This occurs on bearings that failed in the last few samples, such that the MA HI did not reach the threshold. Notice that, during the PHM data challenge, submissions were evaluated on a single point, whereas, here, the evaluation concerns the whole period until the bearing fails.

The proposed model gives better results on average for both metrics and most bearings compared to the baselines (MAPE 714.57 ± 654.10 RMSE 3347.18 ± 2931.54). When comparing the baseline of the full dataset against the truncated one, it is clear that one of the benefits in improving results is the truncation of datasets before training, as this removes data corresponding to long runs in which degradation has not started. The MAPE scores confirm that the HI-derived features have a positive impact on modeling the RUL, especially when the last part of the remaining life is of higher importance. However, it is important to remark that, for some instances, the RMSE of the full model performed better than the proposed model.

#### 4.2.2. Smart Maintenance Living Lab

Table 7 presents the coefficients for SMLL models. Table 8 reports the performance of th RUL model over the run after the HI threshold is surpassed. The performance of the proposed model is better than the baseline trained over the full data (MAPE 215.40 vs. 556.75 and RMSE 659.91 vs. 1796.65). On the other hand, the performance between the truncated baseline and the proposed model is similar (MAPE 209.69 ± 258.13 vs. 214.40 ± 273.62).

These results show that the largest improvement is caused by truncating the datasets to the degradation segment. In this case, the HI-based features likely have no added value when sufficient data are available. Consider that the PHM dataset has only six training bearings, whereas the Smart Maintenance Living Lab consists of 43 bearings. The HI is still beneficial as it allows us to truncate the datasets in an unsupervised approach.

**Figure 21:**
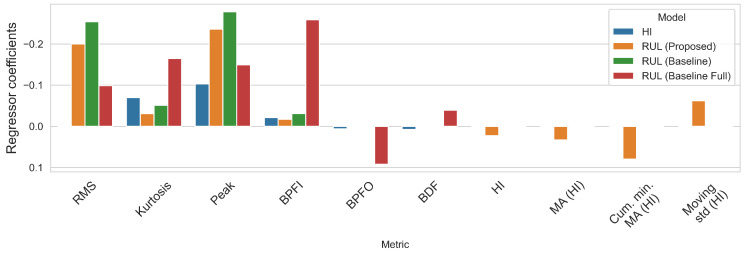
Regressor coefficients for each model on the SMLL dataset.

Furthermore, while inspecting the model coefficients in Figure 21 and Table 5 and Table 7, it is notable how the truncated baseline and the proposed model assign similar coefficients to the relevant features, but the proposed model still uses the HI-derived features.

### 4.3. Applicability

The presented method for the PHM and the SMLL cases was based on bearing-specific fault features. Therefore, its applicability is limited to bearing problems. However, as previously discussed in the Introduction, the methodology concerns a more general problem in which structural learning is applied to temporal features to induce monotonicity. Therefore, the method can be extended to other types of problems as long as temporal features can be derived and these contain some trendability over time, e.g., electrical components or mechanical components of other nature. The technique on its own is suitable for a wide range of PHM problems; for example, the HI model can detect anomalous events as well as degradation onset, and the RUL predictions can assist in scheduling maintenance and preventing failures.

## 5. Future Work

We identify three promising areas for future work:

1. Extending HI properties. This work focused on generating HI by inferring a target with monotonic properties. However, the literature points towards other desirable properties of HI, such as robustness towards noise and sudden changes; trendability, in which the HI is correlated with time; and identifiability, in which the HI is correlated to a sequence of categories [3]. These properties have mathematical definitions and can be easily incorporated into the objective function of the HI model.
2. Transfer learning. As previously commented, the Smart Maintenance Living Lab dataset comprises seven identical setups. In the current approach, all tests were done under the same conditions; therefore, the models are expected to generalize across machines using cross-validation. Although a large dataset can allow generalization across the different setups, a more promising approach is model adaptation, where a new test condition can be learned swiftly using a restricted amount of information. Ideally, a base model could be adapted to a new setup by running as little as a single test.
3. Richer features. The current work presents a limited set of features that can be computed easily. Nevertheless, there is great potential in investigating the presented technique on raw accelerometer data and possibly other sources of information, such as temperature.

## 6. Conclusions

This paper presents a purely data-driven technique for generating HIs. Although its performance cannot be quantified, the index was demonstrated empirically to be a good tool for obtaining insights into the degradation process of the bearing. Additionally, the HI is proven to be beneficial for truncating datasets and restricting the RUL estimation to the degradation phase, as well as serving as a feature for its prediction. The performance of the method is comparable to previous works.

## Figures and Tables

**Table 1:** Summary of the Pronostia dataset.

Condition	Load (N)	rpm	Number of Train Bearings	Number of Test Bearings
1	4000	1800	2	5
2	4200	1650	2	5
3	5000	1300	2	1

**Table 2:** Smart Maintenance Living Lab dataset.

Setup	Load (N)	rpm	Healthy Bearings	Indented Bearings
1	9000	2000	3	6
2			1	7
3			1	2
4			0	5
5			0	6
6			0	6
7			0	6

**Table 3:** Selected features, abbreviations, and equations.

Name	Abbreviation	Equation
Ball pass frequency of inner ring	BPFI	$\frac{nf_{r}}{2}\left\{1+\frac{d}{D}cos\phi \right\}$
Ball pass frequency of outer ring	BPFO	$\frac{nf_{r}}{2}\left\{1-\frac{d}{D}cos\phi \right\}$
Bearing defect frequency	BDF	$\frac{D}{2d}\left\{1-{\left(\frac{d}{D}cos\phi \right)}^{2}\right\}$
Root mean square	RMS	$\sqrt{\frac{1}{N}\sum {\left[x\left(n\right)\right]}^{2}}$
Kurtosis	-	$\frac{\sum_{i}x_{i}^{4}}{{\left(\sum_{k}x_{k}^{2}\right)}^{2}}$
Peak to peak	-	max[x]−min[x]

Where: *n* Number of rolling elements, *f_r_* shaft speed, *ϕ* angle of the load from the radial plane, *d* diameter of the rolling element, *D* the pitch diameter. For BPFI, BPFO, and BDF, the equation provides the frequency of interest from the spectrogram obtained after the FFT of the windowed signal. For RMS, kurtosis, and peak to peak, *x* corresponds to a window of 1 s of the vibration signal.

**Table 4:** Parameter range for each stage.

Stage	HI (Offline)	HI (Online)	RUL
Model	SGD Regressor		
Loss	Squared	Huber	Huber
Regularization	L2	ElasticNet	L2
Alpha	$10^{-4}$ (fixed)	$10^{-4}$–0.1	$10^{-4}$–0.1
Epsilon	-	0.01–0.1	0.01–0.1

**Table 5:** Coefficients for PHM models.

	HI	RUL Models		
	-	Proposed	Baseline Truncated	Baseline Full
BDF_horiz	0.01	−0.11	−0.10	−0.04
BDF_vert	0.01	0.00	0.06	0.03
BPFI_horiz	−0.02	−0.17	−0.21	−0.07
BPFI_vert	−0.01	0.00	0.00	0.05
BPFO_horiz	−0.00	0.00	−0.01	−0.09
BPFO_vert	−0.00	0.00	0.00	−0.03
Kurtosis_horiz	0.00	0.00	0.00	0.00
Kurtosis_vert	0.01	0.00	0.00	0.07
Peak_horiz	−0.03	0.00	−0.09	−0.26
Peak_vert	−0.01	−0.15	−0.21	−0.12
RMS_horiz	−0.02	0.00	0.00	−0.24
RMS_vert	−0.02	−0.03	−0.21	−0.35
condition	-	−0.41	−0.41	−0.44
HI	-	0.02	0.00	0.00
Moving std (HI)	-	−0.34	0.00	0.00

**Table 6:** Results obtained on the PHM dataset. Highlighted in bold the best result per bearing.

		MAPE			RMSE		
Bearing	Samples	Baseline (Full)	Baseline (Truncated)	Proposed Model	Baseline (Full)	Baseline (Truncated)	Proposed Model
Bearing_1_3	649	90.05	53.87	**48.28**	3345.78	**2075.15**	2131.89
Bearing_1_4	337	98.93	**94.34**	96.79	1888.12	**1711.12**	1824.11
Bearing_1_5	200	1411.10	1144.45	**862.94**	12,957.93	3498.21	**3223.30**
Bearing_1_6	803	531.81	371.94	**316.18**	9319.96	**2402.58**	2668.28
Bearing_1_7	200	735.81	846.77	**400.79**	8576.47	2754.55	**2627.02**
Bearing_2_3	1683	269.09	278.16	**251.62**	**6428.16**	7736.51	7949.57
Bearing_2_4	200	1921.50	2183.98	**1888.65**	9965.08	2242.99	**1863.27**
Bearing_2_5	2196	227.05	200.02	**195.93**	**7487.13**	10,329.89	10,599.39
Bearing_2_6	200	1539.52	1796.24	**1552.94**	10,046.48	2206.10	**1856.99**
Bearing_2_7	200	1595.64	1955.84	**1716.00**	8433.22	2010.73	**1698.62**
Bearing_3_3	200	**377.14**	667.66	530.16	3242.77	582.48	**376.51**
**Mean**	-	799.78	872.12	**714.57**	7426.46	3413.67	**3347.18**
**SD**	-	651.69	750.47	654.10	3242.56	2787.76	2931.54

**Table 7:** Coefficients for SMLL models.

	HI	RUL Models		
	-	Proposed	Baseline Truncated	Baseline Full
RMS	0.00	−0.20	−0.25	−0.10
Kurtosis	−0.07	−0.03	−0.05	−0.16
Peak	−0.10	−0.24	−0.28	−0.15
BPFI	−0.02	−0.02	−0.03	−0.26
BPFO	0.01	0.00	0.00	0.09
BDF	0.01	0.00	0.00	−0.04
HI	0.00	0.02	0.00	0.00
MA (HI)	0.00	0.03	0.00	0.00
Cum. min. MA (HI)	0.00	0.08	0.00	0.00
Moving std (HI)	0.00	−0.06	0.00	0.00

**Table 8:** Results obtained on the Smart Maintenance Living Lab dataset. Highlighted in bold the best result per bearing.

		MAPE			RMSE		
Bearing	Samples	Baseline (Full)	Baseline (Truncated)	Proposed	Baseline (Full)	Baseline (Truncated)	Proposed
A8	1136	373.04	116.48	**114.15**	1196.66	246.75	**237.93**
A9	2767	101.46	**61.63**	63.03	1089.83	925.09	**876.15**
A22	1438	234.12	**113.81**	129.88	1779.06	**393.11**	**393.11**
A27	708	1941.62	693.97	**616.20**	3513.95	988.28	**873.19**
A35	1136	314.90	**124.19**	127.57	1601.52	**364.93**	405.23
A37	1337	508.77	126.07	**125.31**	1136.69	314.76	**311.60**
A38	1357	507.39	**194.45**	199.28	1641.67	**450.08**	455.09
A40	512	1971.42	**864.20**	922.92	3274.53	**1150.67**	1316.35
A43	1181	391.77	**138.54**	175.05	2631.96	**569.95**	808.11
A45	1011	259.81	**86.92**	91.11	686.89	229.83	**206.35**
A46	1052	**81.89**	95.44	107.03	382.07	**304.34**	347.29
A47	1949	208.74	**53.04**	59.85	2144.09	**255.93**	338.94
A48	2143	425.51	**87.68**	91.28	1152.33	**549.04**	595.28
A49	1144	200.31	**120.53**	138.76	1729.27	**536.56**	589.85
A50	1137	207.29	**130.05**	137.03	752.06	**279.20**	320.27
A51	4996	112.22	**74.51**	74.98	2041.47	2084.91	**1999.09**
A52	891	284.28	**190.39**	201.09	1546.09	**562.80**	645.03
A53	1691	466.16	92.56	**84.38**	1626.83	**346.05**	352.12
A54	2311	171.72	87.92	**83.84**	819.91	691.35	**684.83**
A55	1689	275.13	103.88	**102.49**	1569.20	340.72	**316.97**
A56	305	1313.47	741.81	**717.04**	1618.53	**865.66**	924.95
A58	2500	178.21	**79.62**	80.30	944.90	**919.43**	921.52
A59	3183	181.70	**67.90**	69.76	1528.33	883.27	**855.61**
A60	1159	587.65	258.17	**235.71**	2456.66	**576.50**	585.48
A62	1100	391.93	143.67	**142.43**	906.63	279.13	**271.51**
A63	4996	258.54	74.98	**71.39**	4874.16	1816.48	**1733.14**
A64	1395	77.69	**65.34**	77.71	413.44	**305.93**	317.50
A66	667	670.99	**301.99**	351.75	2088.31	**655.43**	819.23
A67	322	4510.11	**1302.92**	1450.64	4863.47	**1218.35**	1428.25
A68	2128	151.63	**53.08**	55.30	1287.08	409.15	**376.44**
A69	1066	335.93	**137.94**	149.71	1964.82	**375.91**	417.65
A70	1901	344.84	**88.12**	93.46	1478.86	**339.62**	401.16
A76	2682	255.86	104.67	**97.60**	1021.40	632.21	**595.15**
A77	1325	788.00	253.54	**232.47**	2788.34	567.59	**505.39**
A78	1629	302.29	182.75	**160.46**	1447.02	377.02	**354.76**
A79	1017	1167.27	348.26	**315.29**	3430.86	646.70	**639.33**
A80	1832	392.60	101.31	**95.94**	1782.08	319.09	**288.66**
A81	3891	210.27	105.98	**105.00**	**1061.76**	1597.59	1568.04
Mean	-	556.75	**209.69**	214.40	1796.65	**641.30**	659.91
SD	-	784.64	258.13	273.62	1044.39	435.64	431.29

## Data Availability

Raw data were generated at Flanders Make. Derived data supporting the findings of this study are available from Agusmian Partogi Ompusunggu (Flanders Make) on request.

## Abbreviations

The following abbreviations are used in this manuscript:

AD	Anomaly detection
BDF	Ball defect frequency
BPFI	Ball pass frequency of the inner race
BPFO	Ball pass frequency of the outer ring
CNN	Convolutional Neural Networks
CumMinMa	Cumulative minimum of the moving average
FFT	Fast Fourier Transform
HA	Health assessment
HI	Health index
IIoT	Industrial Internet of Things
LSTM	Long Short-Term Memory
MA	Moving average
MAPE	Mean absolute percentage error
PHM	Prognostics and Health Management
RMS	Root mean square
RMSE	Root mean squared error
RNN	Recursive Neural Networks
RUL	Remaining useful life
SGD	Stochastic Gradient Descent
SMLL	Smart Maintenance Living Lab
